# Delivering HIV services in partnership: factors affecting collaborative working in a South African HIV programme

**DOI:** 10.1186/s12992-016-0228-y

**Published:** 2017-01-13

**Authors:** Geoffrey A. Jobson, Cornelis J. Grobbelaar, Moyahabo Mabitsi, Jean Railton, Remco P. H. Peters, James A. McIntyre, Helen E. Struthers

**Affiliations:** 1 Anova Health Institute, 12 Sherborne Rd, Park Town, Johannesburg, 2190 South Africa; 2 School of Public Health & Family Medicine, University of Cape Town, Cape Town, South Africa; 3 Division of Infectious Diseases and HIV Medicine, Department of Medicine, University of Cape Town, Cape Town, South Africa

**Keywords:** Global health initiatives, Health partnerships, Health systems strengthening, South Africa, Collaboration, HIV

## Abstract

**Background:**

The involvement of Global Health Initiatives (GHIs) in delivering health services in low and middle income countries (LMICs) depends on effective collaborative working at scales from the local to the international, and a single GHI is effectively constructed of multiple collaborations. Research is needed focusing on how collaboration functions in GHIs at the level of health service management. Here, collaboration between local implementing agencies and departments of health involves distinct power dynamics and tensions. Using qualitative data from an evaluation of a health partnership in South Africa, this article examines how organisational power dynamics affected the operation of the partnership across five dimensions of collaboration: governance, administration, organisational autonomy, mutuality, and norms of trust and reciprocity.

**Results:**

Managing the tension between the power to provide resources held by the implementing agency and the local Departments’ of Health power to access the populations in need of these resources proved critical to ensuring that the collaboration achieved its aims and shaped the way that each domain of collaboration functioned in the partnership.

**Conclusions:**

These findings suggest that it is important for public health practitioners to critically examine the ways in which collaboration functions across the scales in which they work and to pay particular attention to how local power dynamics between partner organisations affect programme implementation.

## Background

Global Health Initiatives (GHIs) have become an integral part of the delivery of health services in many low and middle income countries (LMICs) since the mid-1990s [1]. These initiatives include bilateral aid relationships managed by a government agency, such as the President’s Emergency Plan for AIDS Relief (PEPFAR); multilateral initiatives established by global agencies, such as the Global Fund; and public-private partnerships [1].

Several of these GHIs focus specifically on the treatment and prevention of HIV, and have massively increased the resources available for HIV related programmes in the countries most severely affected by the epidemic. This focus has contributed to the significant progress made in reducing HIV transmission and increasing the uptake of antiretroviral therapy (ART) [2]. However, the effects of the increase in resources for HIV-related care on country health systems have been mixed. Positive effects include the rapid scale up in HIV/AIDS services, and associated decreases in AIDS-related morbidity and mortality; increased stakeholder participation in policy and programme development and implementation; improvements in health care infrastructure and laboratory services; capacity building among local health care workers; decreased impacts of HIV on the health workforce; and in some countries, improvements in primary health care services [1, 3, 4]. Alongside these positive outcomes, there have been a range of negative effects on country health systems resulting from the injection of disease-specific resources. Travis et al. [5] summarise these negative effects on country health systems in terms of: duplications, such as multiple drug delivery systems; distortions, such as the creation of a cadre of higher paid health workers; disruptions, through repeated training courses for the same staff; and distractions, through increasing the administrative burden on health care workers.

Some GHIs have responded to these negative effects by adopting a focus on health systems strengthening. The primary purposes of HSS are to improve the overall capacity of local health systems in order to facilitate the sustainability of donor-supported programmes and to promote country ownership of these programmes [6]. Marchal et al. [7] suggest that the HSS activities conducted by GHIs can be broadly categorised as: those that provide inputs or resources; those that reinforce the capacities of health services directly related to the implementation of disease control programmes; and those that focus on the integration of programme activities into general health services.

Health systems strengthening in the context of donor supported programmes requires collaboration at multiple scales, what Ansell [8] terms “compound collaboration”. The scales across which collaboration occurs in partnerships between GHIs and country health systems primarily include: the geographic, the temporal, and the operational [8]. GHI partnerships are likely to be working simultaneously across these multiple scales, depending on the type of activities that they undertake and Ansell [9] notes that the most problematic area of coordination for these partnerships tends to be at the country level, where the actual implementation of health related interventions takes place.

Collaboration, and the factors affecting the relative success of collaborative relationships, has been widely studied, and several authors have proposed conceptual frameworks for understanding these relationships [10, 11]. Thomson et al. [11] suggest five key dimensions of collaboration, namely: governance-the agreed processes for joint decision making; administration-the agreed processes for implementation and management; mutuality-the existence of mutually beneficial interdependencies; norms-the existence of trusting, reciprocal relationships between partners; and organisational autonomy, which requires managing the tension between the interests of individual organisations and the collective interests of the partnership.

In the context of donor funded HIV interventions, the collaborations between partners are also affected by significant power differentials in terms of the relative levels of resources that donors and government departments can access, and the rights to distribute these resources. Brown [12] discusses the tensions that develop in contexts of shared responsibility for delivering health services, noting that whereas governments frequently lack the level of resources available to foreign donors, they remain ultimately responsible for the delivery of health services to local populations. These different types of power (i.e. resource based power and administrative power) play a key role in shaping the implementation of GHI programmes. Resolving the tension between access to resources and the right to deliver them requires constant attention to maintaining effective collaborative relationships [12].

Importantly, the ways in which collaborative partnerships evolve and are managed are context specific and are likely to be affected by local level power differentials between donors and their partners.

There is a lack of research on the practical implementation of the collaborative multi-level HSS efforts implemented by GHIs in partnership with national departments of health. This is an important area of research, as understanding the ways in which these partners negotiate the collaborative relationships necessary to implement HSS programmes will provide important insights into the future implementation of these types of programme. In particular, it is important to understand this process from the perspective of local government partners, as the majority of research to date has focused on the perspective of implementing agencies. This article begins to address this lack by examining South African provincial Departments of Health (DOH) perspectives on their partnership with Anova Health Institute (Anova) which, since 2010, has focused on HSS and technical support in three diverse districts of the country.

### Anova Health Institute’s approach to health systems strengthening

Anova’s HIV and TB related HSS support activities have been primarily funded by USAID/PEPFAR and, in line with PEPFAR’s 2009 shift away from direct service provision, the focus of Anova’s work between 2009 and 2014^1^ was on HSS and the provision of technical support to the South African Government’s HIV response. Support activities were conducted at facility, sub-district and district, and provincial levels. These activities included: training, mentoring and capacity building; support for improving data systems and data management; roving teams which provided district-level support to health facilities; and support for the Department of Health’s (DOH) primary health care re-engineering programme. As such, the implementation of Anova’s HSS interventions in South Africa provided a contextualized, tailor-made approach that relied on collaboration at multiple levels with the Department of Health, including staff at local health care centres and clinics, sub-district and district health managers, and provincial administrators.

## Methods

This study used a qualitative methodology based on structured evaluation interviews conducted between May 2014 and January 2015 by independent consultants with 16 Department of Health district and sub-district level managers and administrators. Interviews were conducted in private rooms at district and sub-district offices of the DOH in Gauteng, Limpopo, and the Western Cape Provinces of South Africa. Participants were purposively selected as part of the independent external evaluation process based on their knowledge of and involvement in the Anova Health Institute HIV and TB support programmes. Interview questions centred on their perceptions of the support provided to DOH staff by Anova at the level at which they worked, and included questions covering each aspect of the support provided, as well as more general questions about the process of working in partnership. The particular types of support provided vary across the geographical range of Anova’s work based on the specific needs in each area. This range includes large metropolitan centres such as Soweto in Gauteng Province, as well as rural health districts of the Western Cape and Limpopo provinces. The evaluation was conducted in these diverse districts in order to capture the complexity of this collaboration.

Interviews were audio-recorded and transcribed; transcripts were loaded into NVivo 10 [13] for analysis. Data were analysed using directed content analysis [14] based on Thomson et al.’s [11] framework and Brown’s [12] analysis of the effects of donor-government power differentials on delivering HIV services in Kenya. Thomson et al.’s [11] framework is useful in the context of this study because the authors propose a comprehensive definition of collaboration based on both a literature review and a systematic analysis of definitions of collaboration across multiple disciplines:

*“Collaboration is a process in which autonomous or semi-autonomous actors interact through formal and informal negotiation, jointly creating rules and structures governing their relationships and ways to act or decide on the issues that brought them together; it is a process involving shared norms and mutually beneficial interactions” *[11].

This definition incorporates five dimensions of collaboration which can be used as a framework for analysing real-world collaborative partnerships. These include: structural dimensions (governance and administration); social dimensions (mutuality and norms); and finally a dimension focusing on agency (organizational autonomy). Importantly, Thomson et al. [11] tested this definition empirically in a large scale survey of organisational directors through which they confirmed the construct validity of the five dimensions of collaboration using structural equation modelling.

Thomson et al.’s [11] dimensions of collaboration are useful, but in the context of collaborations between GHIs and departments of health in low and middle income countries the differences between partners in terms of the types and levels of power they hold, may affect collaborative relationships in unexpected ways. Brown [12] discusses the importance of understanding the different types of power held by partners in health interventions supported by GHIs, and notes that whereas GHI-funded implementing agencies have power in terms of being able to provide resources, exercising this power depends on local health departments facilitating access to patient populations. Brown’s [12] analysis of this issue thus provides a useful frame for examining how the collaboration between Anova Health Institute and the various South Africa provincial Departments of Health functioned.

Initial coding involved repeated reading of transcripts by one author with the aim of classifying interview data in terms of the five dimensions of collaboration identified in Thomson et al.’s [11] framework. During this stage of coding meaningful units of text (basic themes) relating to the collaborative relationship were identified. These basic themes were then classified using Thomson et al.’s [11] five domains of collaboration as organising themes. Basic themes could be included in more than one organising theme. This initial coding scheme was thoroughly reviewed by two other authors, who provided further input and suggestions. Disagreements were resolved through discussion. During the final stage of analysis two authors reviewed the coding scheme specifically focusing on two core analytical questions: “what tensions are evident from the DOH perspective that affect the functioning of collaboration in this relationship?”, and, “how do the power differentials between the two organisations affect the dimensions of collaboration in the partnership?”.

The evaluation research was approved by the University of the Witwatersrand Human Research Ethics Committee, and all participants provided informed consent prior to participation.

## Results

### Governance-agreed processes for joint decision making

Anova’s level of involvement in planning and decision making differed between the three partnership districts. The most active engagement for planning and decision making between the organisations occurred in Limpopo Province, where participants reported that:

“We talk almost every week and they are part of us and everything we do”, (Limpopo Province, Sub District level, Assistant Manager).

“At sub district level we plan together with Anova…whatever we want to do with training et cetera”, (Limpopo Province, Sub District level, Operations Manager).

In the Western Cape, meetings focusing specifically on partnership activities took place on a monthly basis, and Anova representatives were present in DOH management meetings. Although Anova was actively involved in planning and decision making in the Western Cape, DOH participants reported a somewhat lower level of engagement with Anova than in Limpopo Province.

“Our strategic planning is done in an established forum on a monthly basis where we look at issues and plan together operationally”, (Winelands, Sub District level, PHC Manager).

Although the DOH participants noted that Anova was involved in planning processes, this role was viewed in terms of making suggestions, providing input, and giving feedback, rather than actively engaging in the process of formulating plans.

“Anova sits in most of the management meetings to provide input on how to deal with things that go wrong, changes that need to be made. Their suggestions are used and their insights into the difficulties staff members experience are appreciated”, (Winelands, Sub District level, PHC Manager).

“[We should be] creating meeting and sharing spaces for Anova and sub-districts and district to feedback challenges,” (Winelands, District level, Physician).

The least in-depth engagement in planning and joint decision making occurred in Johannesburg, where participants reported holding annual and quarterly meetings where Anova and other partner organisations were able to provide input and assist in dealing with challenges within the HIV and TB programmes. However, participants stated that Anova did not play an active role in developing plans for the local health system.

“We are not doing joint planning. We do it as a city and we will forward the service delivery plan to Anova”, (Johannesburg, Sub District level, Management Staff).

“What we normally do when we have our quarterly reviews is an active participation forum, the partners assist with input to assist with solving problems – a working forum where we meet and evaluate the services”, (Johannesburg, District level, Management Staff).

Participants also noted issues that they felt presented challenges in terms of joint decision making within the DOH-Anova partnership. These included: a lack of communication between sub-district and district levels within the DOH about Anova’s activities; the perception that Anova’s activities were based more on USAID/PEPFAR directives than local health needs; and a lack of coordination between the various activities being undertaken by the DOH, Anova, and other organisations.

### Administration-agreed processes for implementation

The partnership was implemented at health facility level, with Anova providing tailored support to facilities based on their specific needs in terms of HIV and TB prevention, treatment, and care. The main form of support discussed by participants in all three provinces was Anova’s training and mentoring of DOH staff. This included training focused on: nurse initiated and managed anti-retroviral therapy (NIMART); data management and data use; and pharmacy stock control and supply chain management.

“The mentorship model is going well and we are having a skills audit of those being trained on NIMART”, (Limpopo, District level, HAST manager).

“They provide a lot of mentorship and the mentorship is both onsite and offsite, we can contact them on the phone and they physically go and support NIMART nurses and TB management [staff]”, (Johannesburg, District level, Deputy Director).

“Anova needs to continue to train and mentor the data capturers”, (Winelands, Sub District level, HAST Co-ordinator).

In terms of the agreed processes for implementing training and mentoring, training was more formal and relied on the DOH ensuring that staff were available to attend the courses organised by Anova. Mentoring was conducted both formally by teams visiting Anova supported facilities at scheduled intervals, and informally, when DOH staff would contact their Anova mentors to ask for assistance in dealing with a particular patient or problem.

Other areas of implementation discussed by participants included: support for clinic re-organisation, support for stock management, and the direct provision of resources such as computers and air conditioners. These types of support tended to rely on DOH staff requesting direct assistance from Anova.

The main challenge reported by DOH staff in terms of implementation was their perceived inability to influence the type of support provided by Anova, with Anova’s work plan being understood as relating primarily to directives from USAID/PEPFAR, rather than the on-the-ground needs of the health system.

“I feel that what USAID decides [is] what they do, DOH needs are secondary”, (Johannesburg, District level, Medical Manager).

“Sometimes the sub-districts have other needs but Anova has their plans”, (Winelands, District level, Physician).

### Organisational autonomy-managing the tension between individual and collective interests

The fact that responding to the dual HIV and TB epidemics forms only part of the DOH’s work, but is the main focus of Anova’s HSS support interventions, was the key feature of managing organisational autonomy within the DOH-Anova partnership. The ability to provide resources and support meant that Anova faced constant pressure to support DOH activities, as these participants noted:

“We cannot do without Anova, we get gazebos, baby scales et cetera. We cannot live without them, they are very supportive … if I find a clinic there who are not doing the right thing and I phone Anova, I tell them this is what’s happening and they will say ‘we will go there tomorrow and show people how to do the right thing’” (Johannesburg, Sub District level, Manager).

In this vein, participants expressed the desire for Anova to provide a range of support that went beyond the organisation's capacity and mandate, essentially suggesting that Anova and the DOH relinquish a greater degree of autonomy within the partnership in order to work together more closely. For example:

“Anova could be more involved in the strategic planning, finance, supply chain management at district level, there is space and room for them to contribute”, (Winelands, District level, Staff).

The shift in Anova’s work to providing technical support, as opposed to directly providing services, also led DOH staff to reconsider their relative autonomy within the partnership. This was particularly important in areas where Anova had been solely responsible for providing specific services. For example, one sub-district manager stated:

“When we still had Anova staff here there were challenges, as [DOH staff] felt that if Anova [was] here then they [Anova] should take responsibility for ARVs. With the staff leaving it is better, as now the DOH is owning the programme…before we felt conflict and no real ownership” (Limpopo, Sub-District level, Manager).

In some instances, the withdrawal of direct service provision by Anova created an acute awareness of the degree to which the DOH had been relying on Anova to provide specific services:

“We used to have [Anova] people in facilities taking care of PMTCT and now the standard has gone down. The DOH staff took it over but … the Anova staff were in the facility doing the work and DOH staff were not part of the services provided so they felt it was not their function. When Anova people left we tried to bridge the gap but people felt they now had additional work and they felt that government should hire additional people to take over which did not happen”, (Johannesburg, District level, Medical Manager).

The high degree of reliance on Anova by DOH staff was therefore a key challenge requiring specific attention in terms of organisational autonomy when Anova’s support model changed.

### Mutuality-mutually beneficial interdependencies

Thomson et al. [11] note that mutuality is rooted in collaborative partners’ interdependence. This interdependence can be understood both in terms of complementarity, where partners each have unique attributes needed by each other, and in terms of commonalities between organisations due to their shared interests [11]. Both of these features of mutuality are evident in the Anova-DOH partnership.

Complementarity played a key role in sustaining the collaboration, with Anova’s ability to provide specific resources and skills to the DOH. Similarly, the DOH was able to provide the basic infrastructure necessary to access patient populations.In terms of shared interests, for Anova, the organisation’s mission and ongoing funding depended on achieving tangible results, while for the DOH, providing HIV and TB services was core to their mandate as a public health service.

The shift by Anova from direct service provision to technical support had an important effect on the way that mutuality operated in the collaboration in some districts. Specifically, complementarity became a more important feature of the collaboration, as Anova’s support staff provided training to DOH staff. In doing so, the partnership moved beyond simply achieving the shared goals of the collaboration, to developing a more dynamic on-the-ground relationship:

“Previously they [Anova] came and did the work and left. But now they go in and assist in solving problems and they give direction, so we find it more helpful than doing the work People will be left with the skills to do the work. Previously they left without information and now they are imparting information” (Limpopo, Sub-District level, Assistant Manager).

Overall, the types of support provided by Anova were viewed by most DOH respondents as essential to the maintenance of HIV and TB services in their districts and sub-districts. Support for data management, training and mentoring, and physical resources and infrastructure were particularly important from the perspectives of the study participants, and were repeatedly raised as areas requiring ongoing support.

Beyond the basic benefits of managing successful HIV and TB services, DOH staff identified a range of other areas of mutual benefit associated with the partnership. For the DOH, Anova was able to provide an external perspective on health service management and problems affecting particular facilities. In areas where local communities lacked trust in the DOH, Anova’s perceived independence meant that they were able to provide a channel through which levels of trust could potentially be improved.

“An assessment was done about the care we give to [the] community using our primary care assessment tool. The community still has a distrusting relationship with the DOH, so Anova’s role is good in the community for obtaining baseline knowledge for building this trust”, (Winelands, District level, Physician).

### Norms of trust and reciprocity

Maintaining a trusting relationship with the DOH is central to facilitating Anova’s ability to implement its interventions in the health system, and overall there appeared to be a high level of trust in Anova among study participants. The personal relationships between the staff of the two organisations were key to developing and maintaining trust within the collaboration.

“The relationship is good but it revolves around personality. I work very well with the team from Anova…It’s driven by personality not so much what work is being done”, (Johannesburg, District Level, Official).

These relationships were also strengthened by the fact that Anova staff were seen as being readily available when their assistance was needed, and by frequent communication between DOH and Anova staff.

“As a sub district [we] have very open line[s] of communication-it has always been good”, (Johannesburg, Sub District Level, Management).

“We are able to pick up the phone and ask them for anything-we tell each other when we are off the path”, (Winelands, District Level, District Official).

Anova’s willingness to provide support, even when this included roles that were beyond their mandate, further strengthened the level of trust held by the DOH in Anova

“They do service delivery sometimes even if it’s not their role”, (Winelands, District level, Physician).

Communication between the DOH and Anova was therefore central to maintaining a trusting relationship between the organisations, and where participants reported disappointment or uncertainty about Anova’s support, the core problem was generally a lack of clear communication:

“There was a lady that was supposed to come from Anova to do quality improvements but [we are] not sure what has happened, we don't know when she is going to come”, (Johannesburg, Sub-District level, Manager).

“…when we did the baseline study we did it with Anova, we were a team and were actively involved, now we don’t know where the reports have been taken to”, (Limpopo, Sub-District level, Manager,).

## Discussion

Collaboration between GHIs and departments of health in low and middle income countries is an important aspect of implementing health interventions globally. The functioning of these collaborations is shaped by the specific local contexts in which they operate, and the ways in which partnerships are managed in light of the differences between partners in terms of the types power they hold. Anova’s primary source of power within the collaboration was its ability to provide specific resources and support for HIV and TB programmes; while the DOH held power in terms of being able to grant or deny access to patient populations, health care workers, and health care facilities. These different types of power and how they were exercised by each partner affected the various dimensions of collaboration and varied between the different districts in which the partnership operated.

Anova’s power in terms of providing resources was reflected in the degree to which it was involved in the governance domain of the collaboration between the three districts. Governance relates to the agreed processes for joint decision making. In Limpopo Province, where the DOH’s access to resources was heavily curtailed due to the department being under administration between 2011 and 2015 because of financial maladministration, DOH staff reported that Anova was highly involved in planning and decision making compared to the Western Cape and Gauteng, where Anova played more of an advisory role. It is possible that the DOH’s lower level of reliance on external funding and resources in the Western Cape and Gauteng limited Anova’s power to influence planning processes.

Partnership administration, which in this case related primarily to the facility level implementation of interventions, was marked by Anova’s need to manage the tension between responding to local health needs and remaining accountable to their funders. The key finding relating to this domain of collaboration was the DOH’s perception of their inability to influence the types of support received from Anova. While overall DOH participants were satisfied with the support they received, their frustration with not being able to influence the implementation process suggests the potential for a breakdown in the collaboration if the DOH became less reliant on Anova’s assistance. The perception among DOH staff of their lack of influence is therefore critical because of their potential power to deny access to patient populations and health staff and facilities.

Anova’s position of having to juggle its PEPFAR mandate with ensuring that it met DOH needs and expectations also affected the way that organisational autonomy functioned in the collaboration. Thomson [11] discusses organisational autonomy in terms of managing the tension between individual organisational interests and those of the partnership. When Anova was still providing services directly, they were perceived by some DOH respondents primarily as using the DOH as a means to meet their PEPFAR targets. Anova essentially had too much autonomy within the relationship. This became evident when Anova shifted to providing technical support, and DOH staff found themselves responsible for services previously provided by Anova. This shift altered the dynamic between the organisations at the level of implementation, requiring a move from operating in parallel, to a more active engagement between staff of each organisation. In order for Anova to implement their technical support programme, both partners had to relinquish a degree of autonomy. This also had the effect of increasing the DOH’s power within the partnership, as they were now acting as gatekeepers to the health care workers Anova needed to train and mentor in addition to patient populations. This is an important finding because it underscores how changes in funders’ approaches to the focus of global health partnerships affect the functioning of collaborative relationships at multiple levels.

Mutuality within the collaboration was also affected by the shift from direct service provision to technical support. Thomson et al. [11] identify two aspects of mutuality, complementarity and shared benefit. However, in the context of the DOH-Anova collaboration, the complementarity of the two organisations (Anova’s ability to provide resources, and the DOH’s access to Anova’s target populations) was also the source of the power differentials in the partnership. The shift to providing technical support required more explicit negotiation around implementing partnership activities because the move away from service provision required the DOH to take on additional responsibilities and to allow Anova access to its staff for training and mentoring. This effectively increased the DOH’s power within the collaboration, making it essential for Anova to demonstrate the ongoing benefits of the partnership. This partly explains the importance of Anova’s continued provision of some forms of direct support to the DOH even after the shift to technical support. The finding that DOH participants generally felt that the support they received from Anova was of a high standard, and made a valuable contribution to addressing the HIV and TB epidemics, was therefore also key in terms of maintaining the effectiveness of the DOH-Anova partnership.

The final dimension of Thomson’s [11] framework is the development of norms of trust and reciprocity between partners working in collaboration. In the case of the DOH-Anova partnership, the DOH participants generally reported high levels of trust in Anova. The importance of maintaining high levels of trust between the two organisations, and the relative level of work that each put into developing norms of trust, was likely influenced by the overall importance of the partnership to each organisation. Since Anova’s organisational goals depended entirely on the success of the partnership, ensuring that the collaboration remained effective and functional was likely to be more important to Anova than to the DOH. The partnership’s relative importance for Anova versus the DOH meant one would expect that ensuring that the DOH developed and maintained high levels of trust in the partnership would be essential for Anova. This generally appears to be the case based on the DOH respondents, with several noting the ease with which they could ask Anova for assistance and expect rapid results. Equally, Anova’s willingness to undertake work beyond the organisation’s direct mandate is also indicative of the importance of retaining the DOH’s trust. Finally, developing and maintaining personal relationships between Anova and DOH staff were essential, and the role of Anova mentors and managers as key points of contact between the organisations was therefore also critical.

These findings have several implications for collaborations between donor organisations and health departments in low and middle income countries. Firstly, donors need to understand the localised power dynamics that affect how collaboration functions at a grassroots level. For example, Anova’s ability to provide resources gave it a potentially relatively higher level of power in Limpopo Province compared to Gauteng and the Western Cape, and this allowed it to be more involved in the governance aspects of the collaboration in Limpopo. This ability to increase involvement in planning and decision making may provide a key opportunity to strengthen other aspects of collaborative relationships, such as mutuality and norms of trust and reciprocity.

A second important finding of this research is that changes in donor policy at a global level may affect collaborations within countries by shifting the balance of power between health departments and donor organisations. In this case, PEPFAR’s directive to move away from direct service provision increased the DOH’s effective power within the collaboration by making it necessary for Anova to negotiate access to DOH staff and facilities for training and mentoring, rather than simply using the DOH as a conduit to provide patients with services. This change in power dynamics made it critical for Anova to pay specific attention to building and maintaining a trusting relationship with the DOH, as well as ensuring that the DOH continued to value the collaboration. This meant that in some cases Anova needed to continue to provide direct support to the DOH even after PEPFAR’s policy change in order to ensure that the collaboration continued to function.

### Limitations

There were several limitations affecting this research that need to be noted. Firstly, the fact that the evaluation interviews used for this research did not include Anova staff members means that our findings are somewhat one-sided, and it is possible that we would reach different conclusions had we had access to Anova staff members’ views. Secondly, the reliance on a set interview schedule may have limited the extent to which participants discussed the particular aspects of collaboration used in the analysis. Thirdly, the use of purposive sampling limits the external validity of the findings, and while this research may be useful as a frame for understanding collaboration in other contexts, further research is necessary to validate these results. Finally, the authors are employed by Anova Health Institute, and while we made a conscious effort to remain objective, it is possible that our interpretations of the data were skewed by our personal investment in the technical support programme. In spite of this, these findings are novel in terms of the public health literature around GHIs and provide a basis for further, more in-depth research into the functioning of collaborations within this field.

## Conclusion

The ongoing involvement of GHIs in global health, means that collaboration has become increasingly important for the achievement of global health goals. As Ansell [8] notes, collaboration at a global level operates across many different scales. As such, the successful implementation of GHI programmes and interventions requires multiple levels of sub-collaboration, with each level of collaboration being subject to specific contextual power dynamics and processes of negotiation.

This research examined the operation of the DOH-Anova partnership at the level at which implementation is directly managed. While Thomson et al.’s [11] dimensions of collaboration remain relevant in this context, our findings suggest that the particular types of power held by each organisation, and the relative importance of the collaboration to each partner, affected the way that these dimensions operated within the partnership. In the context of the implementation of global health programmes and it is necessary for practitioners to understand the ways in which collaborations function across the scales at which they work; and to pay particular attention to managing the local power dynamics between partner organisations at the level of direct implementation of their interventions.

## Abbreviations

ART: Anti-retroviral therapy
ARV: Anti-retroviral
DOH: Department of health
GHI: Global health initiative
HSS: Health systems strengthening
LMIC: Low and middle income countries
NIMART: Nurse initiated and managed anti-retroviral therapy
PEPFAR: President’s emergency plan for AIDS relief
USAID: United States agency for international development

## Footnotes

1 PEPFAR’s focus on Health Systems Strengthening in South Africa, and hence Anova’s work, changed again in 2015, moving away from broader scale support for health systems and refocusing on HIV with a specific basis in the UNAIDS 90-90-90 targets [15].
